# Marshland restoration benefits Collembola recruitment: a long-term chronosequence study in Sanjiang mire marshland, China

**DOI:** 10.7717/peerj.7198

**Published:** 2019-06-27

**Authors:** Yongjing Dou, Bing Zhang, Xin Sun, Liang Chang, Donghui Wu

**Affiliations:** 1 Key Laboratory of Wetland Ecology and Environment, Northeast Institute of Geography and Agroecology, Chinese Academy of Sciences, Changchun, China; 2 University of Chinese Academy of Sciences, Beijing, China; 3 J.F. Blumenbach Institute of Zoology and Anthropology, University of Göttingen, Göttingen, Germany; 4 Key Laboratory of Vegetation Ecology, Ministry of Education, Northeast Normal University, Changchun, China; 5 Jilin Provincial Key Laboratory of Animal Resource Conservation and Utilization, Northeast Normal University, Changchun, China

**Keywords:** Soybean cultivation, Marshland restoration, Soil properties, Collembola community, Life-history traits

## Abstract

To examine the biodiversity restoration of marshlands after human-induced disturbances, a long-term chronosequence study of Collembola communities was completed that included cultivated treatment (marshes with 15 years of soybean cultivation; CU15), two restored treatments (with 6 and 12 years of agricultural abandonment; RE06 and RE12, respectively), and an intact marshland (IM) as a reference in the Sanjiang Plain, Northeastern China. Changes in the soil properties and Collembola communities under different treatments were analyzed. Soil parameters (i.e., soil organic carbon, available N, P and K, soil moisture) significantly increased from the cultivated treatment to the 6-year agricultural abandoned, and then 12-year agricultural abandoned treatment, indicating that the degraded soil began to recover after agricultural abandonment. The density, species richness and diversity of Collembola in RE12 were significantly higher than in RE06 and CU15, and even surpass the IM, indicating marshland restoration (after 12 years of agricultural abandonment) benefited recruitment and reconstruction of Collembola community. We found soil surface-dwelling Collembola recovered faster than eu-edaphic species, that is probably due to some common traits (i.e., parthenogenesis and fast dispersal) between epi- and hemi-edaphic species. The changes in the vegetation and soil properties during long-term soybean cultivation and agricultural abandonment were the key factors affecting the composition, density, and species richness of soil Collembola.

## Introduction

Worldwide human exploitation and the conversion of ecosystems, including for agricultural practices and urbanization, have caused widespread biodiversity loss and environmental quality decline, leading to the degradation of ecological functioning (Bullock et al., 2011; Butchart et al., 2010; Fischer & Lindenmayer, 2007). This is especially true in wetland ecosystems. Wetlands play a crucial role in ecosystem functioning and services such as the carbon cycle, carbon sequestration, conservation of biological diversity and flood protection (Verhoeven & Setter, 2010; Wang et al., 2012). With the increase in human population and economic growth, wetlands are ubiquitously being intensively used for agricultural purposes (Isunju & Kemp, 2016; Johnston, 2013; Song et al., 2012); more than half of the world’s wetlands disappeared during the 20th century (Davidson, 2014). As ecological restoration can potentially contribute to alleviating this biodiversity crisis, it is being increasingly implemented throughout the world (Benayas et al., 2009; Solis-Gabriel et al., 2017; Zedler & Kercher, 2005). Specially, the rate of abandonment of agricultural lands around the world began to increase exponentially since the 1950s because it was easier to restore natural wetlands lost compared with other human practices (Cramer, Hobbs & Standish, 2008; Mao et al., 2018). The shifts in the composition and structure of vegetation and soil chemical and physical properties caused by these extreme changes in wetlands are fairly well-known, but the knowledge associated with changes in soil biotic communities is still limited because belowground responses are slower than aboveground responses due to the different response rates of plants and soil organisms (Guan et al., 2015; Hedlund et al., 2010). Many studies have explored the response of soil environmental factors and biological diversity to serious agricultural disturbances (Hernández et al., 2017; Wang et al., 2012), but few studies have focused on the dynamics of biodiversity—especially soil biodiversity recovery—after agricultural abandonment, with fewer using a long-term chronosequence approach.

Within the soil, biotic communities play a fundamental role in key belowground processes such as nutrient capture and cycling, energy flow, and soil physical structure-building and control (Jouquet et al., 2006; Murray et al., 2009; De Ruiter, Neutel & Moore, 1998). In turn, interactions between plants and soil can considerably influence soil biota communities (Putten et al., 2013). Among the soil organisms, Collembola are abundant and diverse (Hopkin, 1997). Soil Collembola can be good indicators of soil health due to their close relationship with the soil environment and their sensitivity to habitat disturbance such as agricultural practices (Ponge et al., 2013; Rossetti et al., 2015; Sousa et al., 2006). However, the recovery process of soil Collembola communities in the same location over time after agricultural abandonment is poorly understood.

To recover after agricultural abandonment, organisms either have to survive the disturbance or recolonize disturbed patches from the surroundings (Lindberg & Bengtsson, 2005; Querner et al., 2018). Functional traits refer to the morphological and physiological characteristics that allow species to survive, perhaps differently, in dynamic landscapes. Most studies on how soil Collembola communities respond to succession only focus on their community composition, species richness and diversity, and few studies have used species life-history traits to predict responses to disturbances (Bokhorst, Berg & Wardle, 2017; Gagic et al., 2015; Messier, McGill & Lechowicz, 2010). Thus, in this study, we compared the differences in Collembolan taxonomic diversity and life-history traits in order to analyze and infer mechanisms involved in assembly processes in the wetland recovery process. In soil micro-arthropods, the body length, reproductive strategies, life form, and dispersal ability are probably the key factors regulating survival and colonization after disturbances (Driscoll & Weir, 2005; Lindberg & Bengtsson, 2005; Malmström, 2012).

The Sanjiang Plain in Northeastern China is one of the world’s largest freshwater marsh regions. However, the area of the total marsh region has decreased by 77% due to extensive agricultural exploitation since the 1950s (Wang et al., 2011). The conversion from native marshland to agricultural land caused significant changes in the vegetation and soil properties, leading to a sharp decrease in biodiversity (Liu, Lv & Zhang, 2004; Wang et al., 2012). Fortunately, a large number of marshland protection and restoration programs have been implemented since the 1990s due to the growing awareness of the important ecological function of marshlands. However, the effects of different restoration years on the wetland species composition and diversity have rarely been reported. Therefore, we used a chronosequence approach to study the effects of marshland restoration (after 0, 6, and 12 years of agricultural abandonment) on the taxonomic structure, diversity, and life-history traits of Collembola, along with soil physicochemical properties. The use of the chronosequence method enabled the observation of the dynamics of long-term changes in marshland succession after tillage.

Within this context, we aimed to: (1) elucidate the changes in soil Collembola communities in marshes that have experienced different periods of agricultural abandonment and (2) investigate the recovery pattern of soil Collembola after agricultural abandonment. We hypothesized that: (1) the density, species richness, and diversity of Collembola increase after agricultural abandonment, and (2) surface-dwelling (epi- and hemi-edaphic) species recover faster than soil-dwelling (eu-edaphic) species in the soil profile.

## Materials and Methods

### Site description and experimental design

The study was conducted at the Sanjiang Mire Marshland Experimental Station at the Chinese Academy of Sciences (47°35′N, 133°31′E) in the Sanjiang Plain, China (Fig. S1). The station is at an altitude representative of the natural freshwater marshland habitats of the Sanjiang Plain. The study site has a temperate continental monsoon climate with a mean annual air temperature of 2.52 °C, precipitation of 558 mm (more than 65% falls in July and August), and a frost-free period of 125 days (Zhang et al., 2014). The monthly mean temperature was −20 °C in January and 22 °C in July (Song et al., 2009).

Based on the marshland distribution and the present conditions of the farmland, four treatments, including one cultivation treatment, two restoration treatments and one reference, were established. The cultivation treatment (CU15) had been used for soybean planting since 1995 (CU15). The two restoration treatments (RE06 and RE12) were established on soybean fields that had been agriculturally abandoned since 2004 (RE06) and 1998 (RE12), respectively. Undisturbed natural marsh was chosen as intact marshland (IM). The dominant vegetation of the intact marshland and restored marshland both were *Calamagrostis angustifolia*.

Soil samples were taken on June 15, August 15, and October 15 yearly from 2010 to 2012. A total of 10 replicates were randomly selected from each sample site (50 × 50 m^2^), and all soil samples (10 × 10 × 10 cm^3^) were carried in polythene bags to the laboratory. We recognize that these 10 samples per site are not true replicates for the sites, but consider that the pooled sample is sufficiently large to be representative of each treatment. Collembola were then extracted using a Tullgren apparatus (Van Straalen & Rijninks, 1982). The extracted Collembola were preserved in 95% ethanol and identified to species or assigned a morphospecies according to the keys developed by Bellinger, Christiansen & Janssens (1996–2012) and Yin (1992, 1998) using a light microscope.

### Analysis of soil physicochemical properties

The soil physicochemical properties were analyzed based on the methods described in Lu (2000). Soil moisture (SM) content was measured by oven drying (105 °C, 48 h). Soil pH was measured in a soil water suspension (1:2.5 *w*/*v*) with a pH meter. Soil organic carbon (SOC) was determined by dry combustion using a C/N analyzer (LECO Corporation, St. Joseph, MI, USA). The available nitrogen (AN), available phosphorus (AP), and available potassium (AK) were quantified using the alkaline hydrolysis diffusion method, Bray-1 method, and ammonium acetate extraction method, respectively.

### Statistical analysis

We calculated the number of individuals for all identified species and morphospecies for each soil sample. We then used the number of individuals to account for the total amount of captured individuals to classify the quantitative degree of each taxon: the number of individuals that accounted for more than 10% of the total number of captured individuals was dominant species, the number of individuals that accounted for 1.0–10.0% were common species, and the number of individuals that accounted for less than 1% were rare species (Gao et al., 2016). We also explored several parameters to monitor the composition and biodiversity changes in the different wetlands. First, we determined the collembolan density (number of individuals per unit area) and the species richness (number of taxa) for each soil sample. Then we used Shannon-Wiener diversity (*H′*) (Shannon & Warren, 1949) to evaluate soil collembolan diversity in each habitat.

In order to determine whether marsh cultivation and restoration induced changes in the life-history traits of Collembola, we selected four traits that have previously explained shifts in Collembola species composition (Bokhorst et al., 2012; Makkonen et al., 2011; Vanhee et al., 2017; Widenfalk et al., 2015). The selected traits were body length of adults, life form, reproductive mode, and dispersal traits. Table S1 provides definitions and ecological significance (Berg et al., 1998; Ponge et al., 2006; Rusek, 2007). Trait values (Table S2) were obtained from various literature sources (Chen & Christiansen, 1996; Li, Hua & Chen, 2008; Palaciosvargas & Martinez, 2014; Vanhee et al., 2017; Yun & Gao, 2015). We calculated the community weighted mean (CWM) trait values for each of four traits according to Garnier et al. (2004), weighing species traits in each sample by the relative abundance. For each sample, the CWM trait values were calculated as follows:

(1) $${\rm{CWM}} = \sum\limits_{i = 1}^n {pi} \times {\rm{T}}pi$$

where *pi* is the relative abundance of collembolan species *i*, T*pi* is trait score of species *i*, and *n* is the number of species included in the calculation.

Means and standard errors were calculated for soil parameters and the Collembolan indices of samples from each treatment. One-way analysis of variance (one-way ANOVA) was used to test the difference significance of the soil properties among treatments. In addition, repeated measures ANOVA was used to test for significant differences in density, species richness, Shannon-Wiener diversity, and CWM trait values among different treatments. The significance of post hoc pairwise comparisons were determined using Fisher’s least significant difference (LSD) tests under significance levels of 0.05. In addition, a Spearman correlation analysis was conducted to identify correlations between the soil properties and the response variables of the Collembola, including the abundance of taxa, species richness, and diversity. All statistical analyses were performed using the software package IBM SPSS statistics 22.0 for Windows (IBM SPSS Inc., Armonk, NY, USA).

The Collembola community was further analyzed using the software package CANOCO Version 5.0 for Windows. To test the relationships between Collembola community and recovery years, we performed analyses using redundancy analysis (RDA) based on the species abundance. The soil properties and species abundance were standardized via log transformation before analysis. The significance of the RDA results was determined using a permutation test (499 permutations). We also used an interactive forward-selection RDA (using the “manual selection of environmental variables” option in CANOCO 5.0) to test which variable among the soil properties significantly influenced the Collembola composition. The selection procedure was stopped when the next factor to be added was no longer significant (Xu et al., 2017).

## Results

### Soil physicochemical properties

All soil properties were significantly different among treatments (Table 1). Generally, SOC, available N, P and K, and SM significantly increased from the cultivated treatment to the 6-year agricultural abandoned, and then 12-year agricultural abandoned treatment (Table 1). By contrast, soil pH value in the intact marshland was significantly lower than the cultivated and agricultural-abandoned treatments (Table 1).

**Table 1:** Mean values (mean ± SE) and significance tests of soil properties across all sites.

	pH	SOC (%)	AN (mg/kg)	AP (mg/kg)	AK (mg/kg)	SM (%)
IM	5.01 ± 0.11^b^	8.13 ± 0.70^a^	562.80 ± 96.55^a^	53.73 ± 2.20^a^	404.13 ± 60.40^a^	32.87 ± 0.28^b^
CU15	5.28 ± 0.03^a^	2.32 ± 0.13^c^	211.68 ± 30.22^b^	21.20 ± 1.83^b^	69.12 ± 7.75^c^	24.64 ± 0.65^c^
RE06	5.40 ± 0.02^a^	4.90 ± 0.20^b^	351.12 ± 17.90^b^	23.39 ± 3.34^b^	167.39 ± 8.15^b^	32.69 ± 0.64^b^
RE12	5.36 ± 0.04^a^	5.74 ± 0.80^b^	403.20 ± 49.70^a^	44.47 ± 1.97^a^	298.43 ± 31.72^a^	44.59 ± 2.82^a^
ANOVA	0.002	<0.001	0.001	<0.001	<0.001	<0.001

**Notes:**

IM, intact marshland; CU15, soybean soils cultivated for fifteen years; RE06, agricultural abandoned for six years; RE12, agricultural abandoned for twelve years; SOC, soil organic carbon; AN, available soil N; AP, available soil P; AK, available soil K; SM, soil moisture. Different superscript lowercase letters within the same row indicate significant differences between treatments based on the least significant difference (LSD) test (one-way ANOVA; *p* < 0.05).

### Community composition of collembola

In total, 8,171 individuals and 28 species, with 4,669/25, 5,132/28, 8,539/28 of Collembola (Table S3) in the IM, CU15, RE06 and RE12 fields, respectively, were identified. Five Collembola species—*Bourletiella* sp. 2, *Entomobrya* sp. 2, *Tomocerus nigrus*, *Desoria* sp. 3, and *Folsomia* sp. 3—disappeared in the cultivated fields, and all of these species recovered in the restored wetlands (Table S3). *Allonychiurus songi* was the dominant species at all sites (Table S3). *Desoria* sp. 1, *Desoria* sp. 2, *Desoria* sp. 4, *Hypogastrura* sp. 1, and *Hypogastrura* sp. 2 were rare species in the cultivated fields but were common species in the marshlands (Table S3). *Entomobrya* sp. 1 and *Folsomides* sp. 1, which were absent in the IM soils, occurred in the cultivated and restored fields. *Entomobrya* sp. 3, *Isotomodes* sp. 1, and *Tullbergia* sp. 1 were absent in the restored fields whereas *Hypogastrura* sp. 3 only appeared in these fields (Table S3).

**Figure 1:**
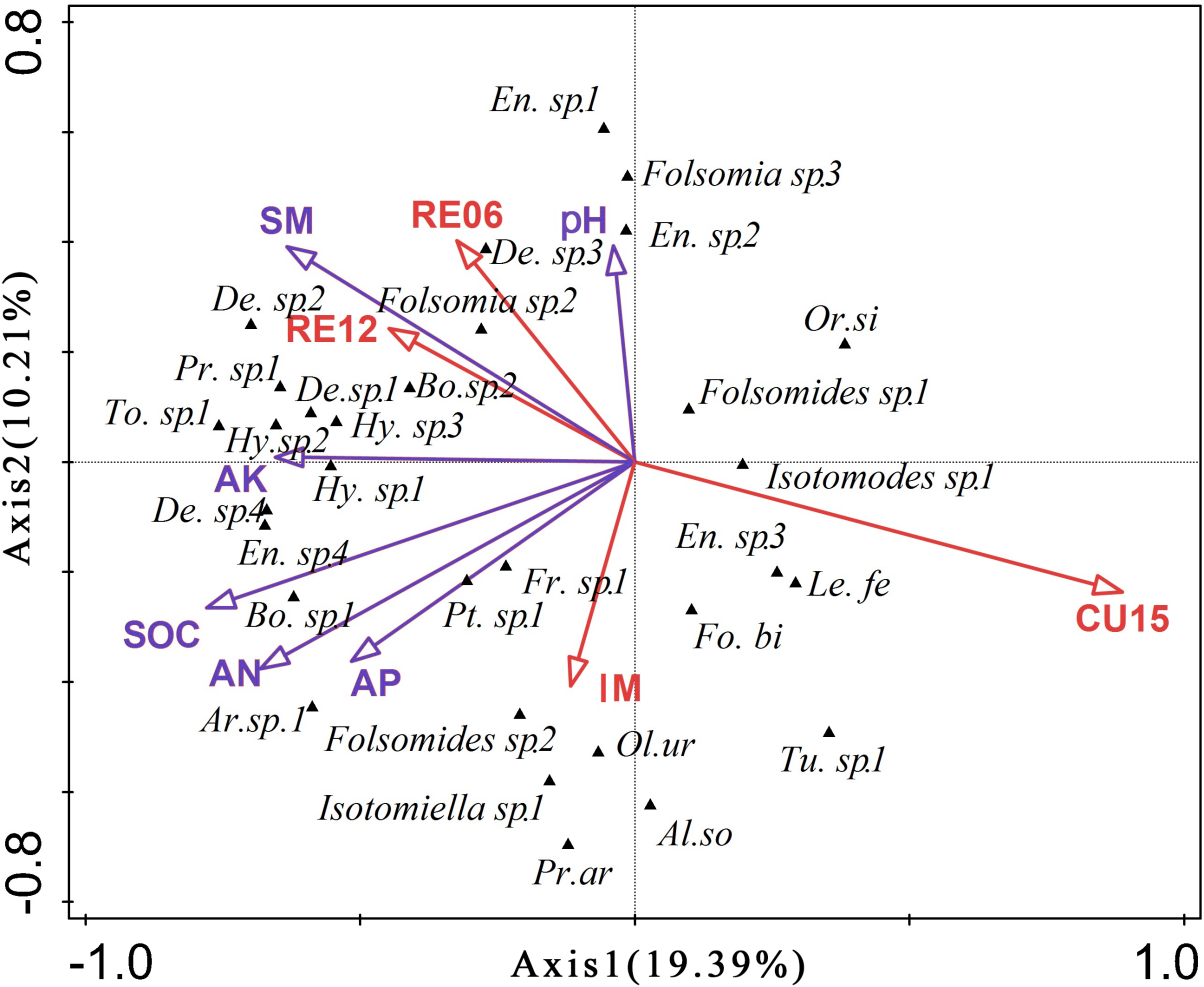
Redundancy analysis (RDA) diagram for soil Collembola communities across all sites. *Bo*. sp. 1, *Bourletiella* sp. 1; *Bo*. sp. 2, *Bourletiella* sp. 2; *En*. sp. 1, *Entomobrya* sp. 1; *En*. sp. 2, *Entomobrya* sp. 2; *En*. sp. 3, *Entomobrya* sp. 3; *En*. sp. 4, *Entomobrya* sp. 4; *Le. Fe, Lepidocyrtus felipei*; *Or. Si, Orchesellides sinensis*; *Pt*. sp. 1, *Ptenothrix* sp. 1; *To. Ni, Tomocerus nigrus*; *De*. sp. 1, *Desoria* sp. 1; *De*. sp. 2, *Desoria* sp. 2; *De*. sp. 3, *Desoria* sp. 3; *De*. sp. 4, *Desoria* sp. 4; *Fo. Bi, Folsomia bidendata*; *Fr*. sp. 1, *Friesea* sp. 1; *Hy*. sp. 1, *Hypogastrura* sp. 1; *Hy*. sp. 2, *Hypogastrura* sp. 2; *Hy*. sp. 3, *Hypogastrura* sp. 3; *Pr*. sp. 1, *Proisotoma* sp. 1; *Ar*. sp.1: *Arrhopalites* sp.1; *Al. so*: *Allonychiurus songi*; *Ol. ur*: *Oligaphorura ursi*; *Pr. ar*: *Protaphorura armata*; *Tu*. sp. 1: *Tullbergia* sp. 1.

The first two RDA axes together explained 29.60% of the variation in the Collembola community (*p* = 0.032) (Fig. 1). The first axis explained 19.39% of the total variance in species composition and represented mainly SOC, AN, AK, and SM gradient. The second axis which accounted for 10.21% of the total variance was mainly represented by SM and soil pH. The interactive forward selection showed that SOC (*p* = 0.006) significantly affected soil Collembola structures, explaining 15.7% of the total variation.

### Density, richness, and diversity of Collembola

**Figure 2:**
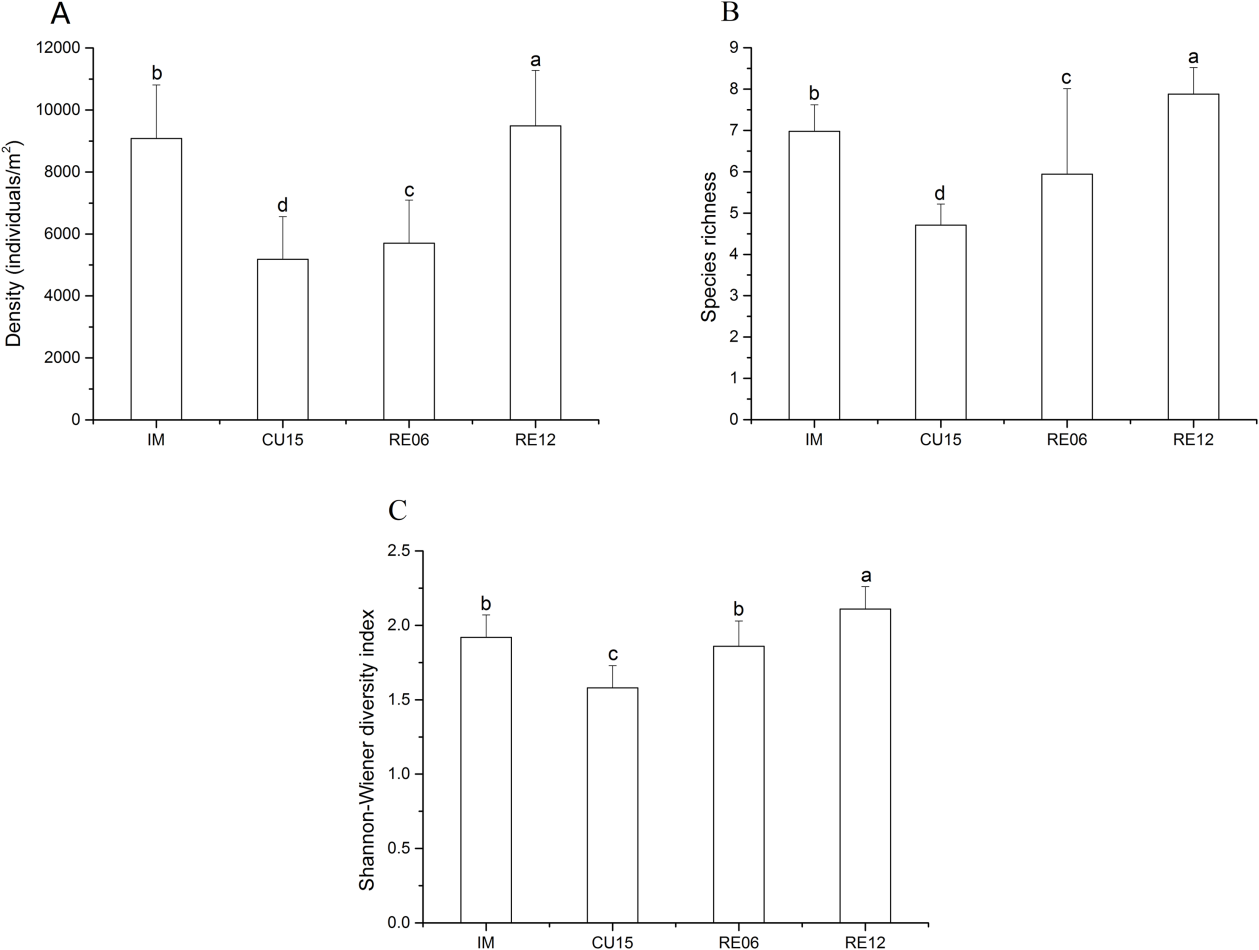
Effects of marshland management on density (A), species richness (B), and Shannon-Wiener diversity index (C) (mean ± SE) of Collembola across all sites. Different letters indicate a significant effect among habitats based on LSD test (repeated measurement ANOVA; *p* < 0.05).

The density and species richness showed a significantly increasing trend in the order of CU15, RE06, IM, and RE12 (Figs. 2A and 2B). The density of the hemi-edaphic Collembola species increased significantly with the length of agricultural abandonment (Table S4). The Shannon-Wiener diversity value was significantly higher in the RE12 than in the IM, RE06, and CU15 soils, whereas no significant variations were found between the IM and RE06 sites (Fig. 2C). The Spearman correlation analysis showed that the total abundance was positively correlated with soil SOC, AN, and AP (Table 2). The epi-edaphic species abundance was positively correlated with soil pH and SM (Table 2). Hemi- and eu-edaphic species abundance were positively correlated with soil SOC, AN, and SM (Table 2). Species richness was positively correlated with soil SOC and AN.

**Table 2:** Spearman correlation coefficients of Collembola community variables with soil pH, SOC, AN, AP, AK, and SM.

	pH	SOC	AN	AP	AK	SM
Total abundance	−0.011	0.508*	0.506*	0.447*	0.274	0.226
Epi-edaphic species abundance	0.621**	0.066	0.084	−0.079	−0.050	0.462*
Hemi-edaphic species abundance	0.331	0.481*	0.465*	0.397	0.395	0.598**
Eu-edaphic species abundance	0.313	0.564**	0.537*	0.380	0.396	0.582**
Species richness	0.109	0.481*	0.449*	0.432	0.349	0.412
Shannon-Wiener diversity index	0.178	0.395	0.377	0.346	0.299	0.362

**Note:**

Values in bold indicate significant correlations (*p < 0.05, **p < 0.01).

**Figure 3:**
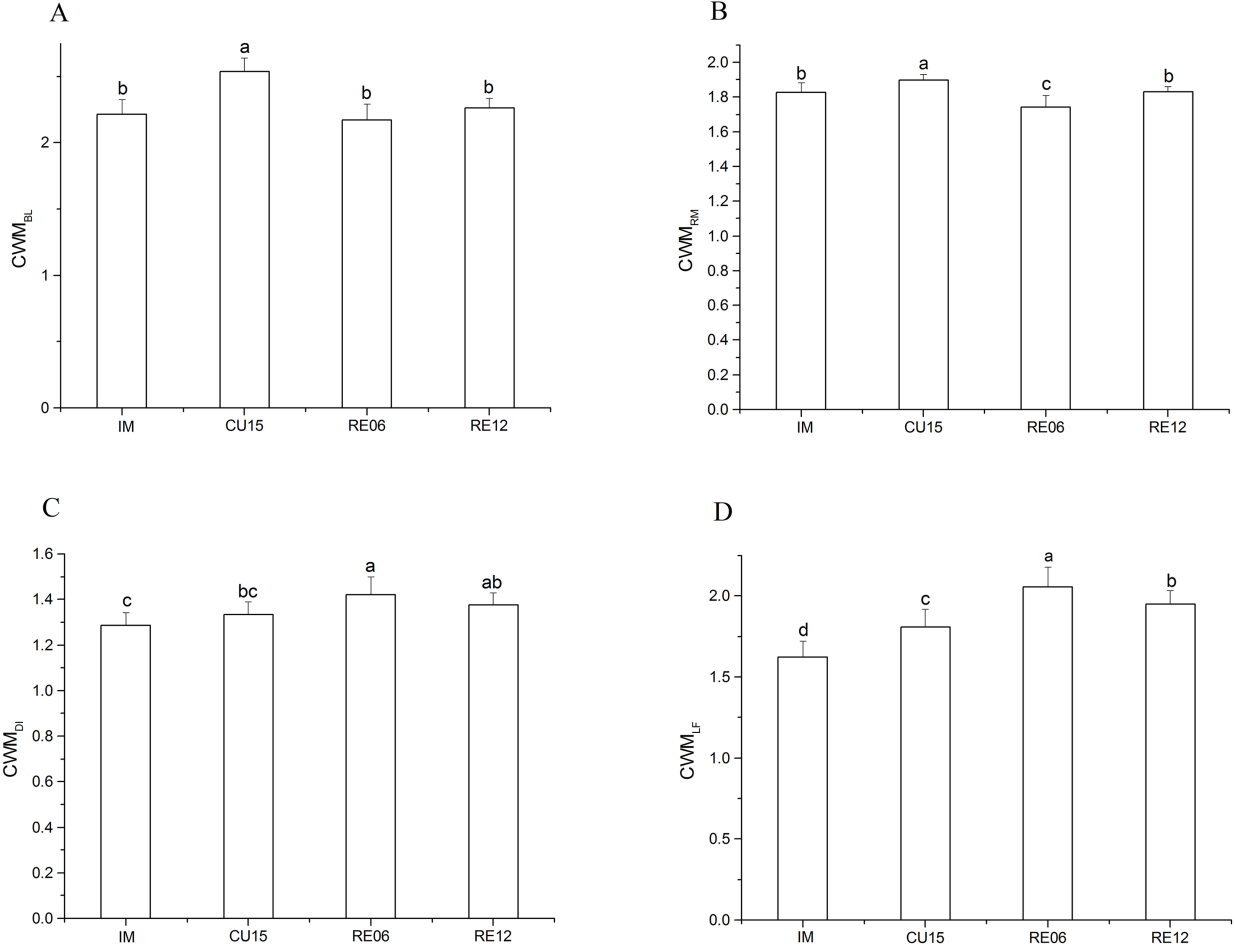
Effects of marshland management on community weighted mean (CWM) body length (A), reproduction (B), dispersal (C), and life form (D) (mean ± SE) of Collembola across all sites. Different letters indicate significant differences among treatments based on the least significant difference (LSD) test (repeated measurement ANOVA; *p* < 0.05).

Markedly significant effects on all CWM trait values were affected by different periods of agricultural abandonment. The CWM_BL_ (*F* = 14.94, *p* < 0.001) of the cultivated treatment (CU15) was higher than that of the IM and restored treatment (Fig. 3A). The CWM_RM_ was highest for the CU15 field and lowest for the RE06 field (*F* = 7.53 *p* < 0.001) (Fig. 3B). The CWM_DI_ was significantly higher for RE06 than for the IM and CU15 fields (*F* = 6.05, *p* = 0.002) (Fig. 3C). The CWM_LF_ for RE06 was significantly higher than that for RE12, and for RE12, this index was higher than for the cultivated treatments and the IM (*F* = 27.54, *p* < 0.001) (Fig. 3D).

## Discussion

### Recovery of soil properties

In our study, the soil nutrient levels, such as SOC, AN, and AP, decreased after marshland conversion to cultivation and increased after agricultural abandonment. These results were consistent with the findings of Xu et al. (2017). We speculated that two factors potentially contributed to the loss of nutrients after reclamation. One is that the grain and straw were harvested and removed off-site, greatly reducing the input of the organic matter which then decreased. Another reason is that wetland soil was water-saturated over long periods of time, and the organic matter decomposed more slowly and even gradually accumulated because of the anaerobic conditions (Compton & Boone, 2000). Once converted to agriculture, the removal of vegetation for cultivation and the exposure of the soil to the wind and sun increased the aeration and soil temperature, leading to increased soil organic matter decomposition. Besides, tillage generally decreased soil organic matter due to erosion and disruption of the physical, biochemical, and chemical mechanisms of SOM stabilization, and increase soil available C, K, and P leaching (Wei et al., 2014). In contrast, the increase of nutrients found in the restoration treatments could be ascribed to the increased litter input after agricultural abandonment, as the straw and grains were no longer removed from the marshland system. In addition, SOM and available nutrients can generally accumulate after the cessation of cultivation. In short, our findings strongly indicate that the cultivation of marshland led to soil degradation and that agricultural abandonment could improve soil quality.

### Recovery of the Collembola community

In line with our first hypothesis, we found the density, species richness, and Shannon-Wiener diversity of Collembola sharply decreased in CU15 fields and increased in the restoration sites. Furthermore, the RDA result showed that the first axis separated the cultivated treatments from the IM and restored treatments, and the IM and restored treatment were separated by the second axis. They all indicated that marshland management significantly affect the community structure of Collembola. For instance, five species of Collembola which disappeared in the cultivated fields all recovered in the restored wetlands; however, the community structure of Collembola in restored marshland were also different from intact marshland. *Folsomia bidendata* and *Tullbergia* sp. 1 were more frequent identified in intact marshland soils while *Entomobrya* sp. 4, *Folsomides* sp. 2 and *Folsomia* sp. 2 were more frequent in restored marshland soils. *Entomobrya* sp. 3, *Isotomodes* sp. 1 and *Tullbergia* sp. 1 existed in the intact marshland soils while disappeared in the restored fields. *Hypogastrura* sp. 3 which was the new introduced species only existed in the restored marshland. We may infer that marshland restoration (after 12 years of agricultural abandonment) can significantly promote the recovery of the species diversity of Collembola; however, it might be difficult to restore to its original state once the composition of the marshland Collembola community has been altered by tillage. That is to say, natural restoration can recruit and reconstruct Collembola community composition.

In the present study, we found that the Collembola community was significantly affected by the SOC. This supports Pipan & Culver (2013) assumption that the SOC content is an important factor limiting the occurrence of organisms in shallow subterranean habitats, as the SOC could provide soil fauna with abundant food resources. In addition, the correlation analysis of the Collembola variables with environmental variables exhibited the significant positive correlation of AN and AP with total abundance and species richness of the Collembola. The reason for this relationship may be the fact that N and P can indirectly influence the habitat and diet of Collembola (Setälä, Marshall & Trofymow, 1995). Compared with RE06, the soil parameters of the RE12 were more similar to those of IM, demonstrating that 12 years was enough time for the species composition or diversity of Collembola to fully recover from a strong disturbance such as long-term soybean cultivation. Thus, in present study, we concluded that the soil Collembola communities were influenced by multiple soil parameters and were most affected by the SOC and nutrients that provide food and habitat for organisms (Birkhofer et al., 2012; Yin et al., 2018).

In support of our second hypothesis, we found higher CWM life forms of Collembola in the restoration sites, which is indicative of a community that has a greater contribution of surface-dwelling species. This is consistent with the observed increasing trend in the density of hemi-edaphic species after agricultural abandonment. This response of Collembola to marshland recovery is likely due to changes in the vegetation and soil properties after agricultural abandonment. Sabais, Scheu & Eisenhauer (2011) found that Collembola density and diversity increased with an increase in plant species and plant functional richness. Vegetation offers ecological links between the aboveground and belowground biota, affecting the diversity and quality of the litter that serves as a habitat and food resource for Collembola (Hopkin, 1997; Wardle, 2013). Particularly, the density of hemi-edaphic species living in upper soil and litter continue to increase and are likely influenced by the increased plant diversity and litter input after agricultural abandonment, as the straw and grains are no longer removed from the ecosystem. In the present study, we found the lowest CWM life form for Collembola in the intact marshland, which indicated the greater contribution of eu-edaphic species. This observation could be explained by the fact that the intact marshland habitat has thicker litter layers, allowing the maintenance of a higher resource quality and more stable conditions in terms of moisture and temperature, which is a preferable environment for soil-dwelling species (Berg & Bengtsson, 2007; Heiniger et al., 2015). Thus, the CWM trait of the life forms at the sites showed a response to the site gradient and soil properties, with a higher proportion of epi-edaphic and hemi-edaphic Collembola species at the recovery sites compared with the intact marshland and soybean fields. Similar results were reported by Da Silva et al. (2016).

In this study, the mean traits of the Collembola community differed remarkably between the cultivated and restoration treatments. We found CWM_BL_ increased at the farmland sites, which is indicative of a community that the decline in smaller size individuals of collembolan and the community turns to a *K*-strategy. However, the CWM_BL_ at the restoration sites declined to similar level as the control sites, it means the body length of collembolan go back to the original levels after marshland restoration. In general, parthenogenetic species were more ready to occupy new territory and became an invasive one, because parthenogenesis is an advantageous trait for colonization (Norton, 1994) which may facilitate the establishment of populations from very few individuals (Lindberg & Bengtsson, 2005). In our study, the peak CWM_RM_ was found in the CU15 fields and the minimum CWM_RM_ occurred in the RE06 fields. It may be caused by two reasons. First, the natural disturbance caused by flooding and dry-wet alternate action in marshland was greater than the human-caused disturbance in farmland due to the consistent state of dry field. Second, abundant food resources were conducive to the rapid reproduction of parthenogenetic groups. Compared with farmland, the food resources in marshland were relatively abundant because the litter always exit. So we considered the parthenogenetic taxa recovered faster than sexual individuals. Lindberg & Bengtsson (2006) found a tendency for the more mobile species of arthropods to recover faster than slow moving species. This was also found to be true in our study, where communities in areas undergoing restoration treatments were faster-moving species. Species with a high dispersal ability have an advantage in the colonization of habitats after disturbances (Burrows & Sutton, 2008) and the traits associated with dispersal were selected by land-use change in this prior study. In the present study, we use life history traits to reveal the recovery patterns in Collembola community after different periods of agricultural abandonment. Compared with taxonomic indices, the use of species traits is genetic and reduces environmental dependency, allowing for widely application of monitoring studies (Da Silva et al., 2016; Pey et al., 2014).

## Conclusions

Our study showed that the density, species richness and Shannon-Wiener diversity of Collembola increased in the restoration sites. The results indicated that marshland restoration (after 12 years of agricultural abandonment) benefited recruitment and reconstruction of the community composition of Collembola. It also significantly promoted the recovery the species diversity of Collembola which mainly due to the improvement of soil properties. We also observed that surface-dwelling species recover faster than soil-dwelling species, maybe due to some common traits (i.e., parthenogenesis and fast dispersal) between epi- and hemi-edaphic species. Both taxonomic indices and functional indices based on life history traits revealed different aspects of community assembly. Therefore, we believed that the combination of both taxonomic indices and life history traits of Collembola is a promising tool for monitoring and understanding the mechanisms underlying community patterns after human-induced disturbances.

## Supplemental Information

10.7717/peerj.7198/supp-1 Supplemental Information 1 The location of the Sanjiang Mire Marshland Experimental Station, Chinese Academy of Sciences (CAS).

10.7717/peerj.7198/supp-2 Supplemental Information 2 Species traits used in the analyses, their definitions and ecological significance, and the scores of each trait for species observed in the samples. Epi-edaphic: surface-dwelling, hemi-edaphic: litter-dwelling, eu-edaphic: soil-dwelling. References: ^a^ (Berg et al., 1998); ^b^ (Ponge et al., 2006); ^c^ (Rusek, 2007).

10.7717/peerj.7198/supp-3 Supplemental Information 3 Functional trait modalities of the collembolan species collected during the study. 1. Body length: 1: very small (< 1 mm); 2: Small (from 1 to 2 mm); 3: Medium (from 2 to 3 mm); 4: Large (> 3 mm).
2. Reproduction type: 1: parthenogenetic; 2: sexual.
3. Dispersal: 1: slow; 2: fast.
4. Life form (Bokhorst et al., 2017): 1: eu-edaphic; 2: hemi-edaphic; 3: epi-edaphic.

10.7717/peerj.7198/supp-4 Supplemental Information 4 Total number of Collembola of all sampling times across all sites. “+++” stands for dominant species, with individuals accounting for more than 10% of the total individuals; “++” stands for common species, with individuals accounting for 1% ∼ 10%; “+” stands for dominant species, with individuals accounting for less than 1%.

10.7717/peerj.7198/supp-5 Supplemental Information 5 Effects of marshland management on density (mean ± SE) of Collembola with three typical life-forms (Epi-, Hemi- and Eu-edaphic). Capital letters indicate a significant effect among habitats and lowercase letters indicate a significant effect in the same habitat based on LSD test (repeated measurement ANOVA; P<0.05). Ind/m^2^: individuals/m^2^.

## Additional Information and Declarations

### Data Availability

The following information was supplied regarding data availability: The functional trait modalities of the collembolan species collected during the study are available in Table S1.
