# Supplementary figures and images for: Marshland restoration benefits Collembola recruitment: a long-term chronosequence study in Sanjiang mire marshland, China
Source: PeerJ. 2019 Jun 27;7:e7198. doi: 10.7717/peerj.7198 (PMC6599674; doi:10.7717/peerj.7198)

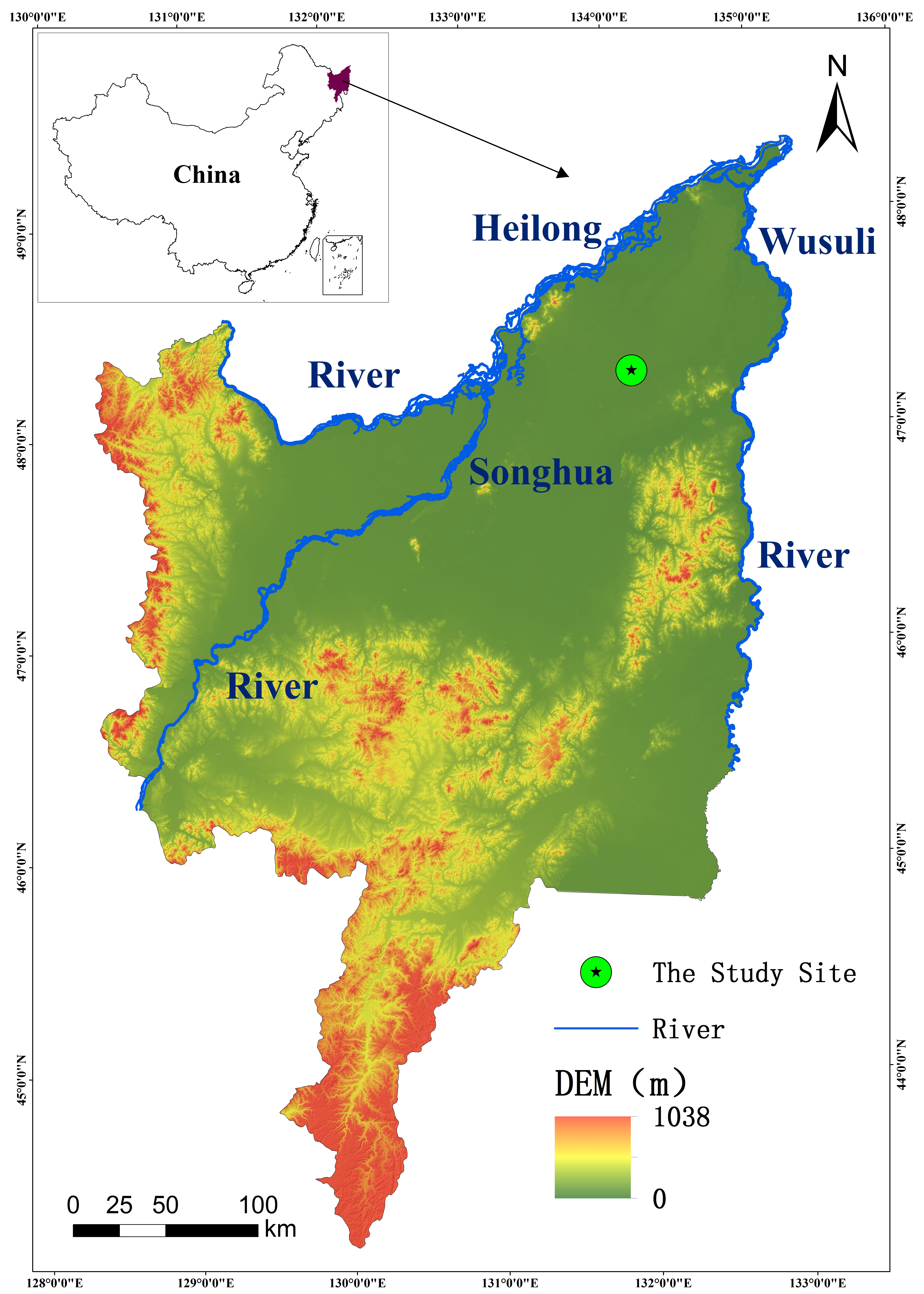

Supplement: Supplemental Information 1 [file peerj-07-7198-s001.png]
