# Supplementary material for: Marshland restoration benefits Collembola recruitment: a long-term chronosequence study in Sanjiang mire marshland, China
Source: PeerJ. 2019 Jun 27;7:e7198. doi: 10.7717/peerj.7198 (PMC6599674; doi:10.7717/peerj.7198)
Supplement: Supplemental Information 2 — Epi-edaphic: surface-dwelling, hemi-edaphic: litter-dwelling, eu-edaphic: soil-dwelling. References: a (Berg et al., 1998); b (Ponge et al., 2006); c (Rusek, 2007). [file peerj-07-7198-s002.docx]

| **Traits** | **Definition** | **Ecological Significance** | **Score** |
| --- | --- | --- | --- |
| Body length | Maximum length from head to tip of abdomen (in mm) | Connected dispersal ability, life form, ecophysiology ^a,b^ | 1: <1 mm  2: 1–2 mm;  3: 2–3 mm;  4: >3 mm |
| Reproduction mode | Reproduction type | Reproduction | 1: Parthenogenetic ;  2: sexual |
| Dispersal | The ability of move | dispersal, predator avoidance | 1: slow;  2: fast |
| Life form | Trait complex composed of number of ommatidia, intensity of coloration, and length of furcula ^c^ | Proxy for vertical stratification, ecophysiology and dispersal ability ^b,c^ | 3: Epi-edaphic *;  2: Hemi-edaphic;  1: Eu-edaphic |

**Table S1.** Species traits used in the analyses, their definitions and ecological significance, and the scores of each trait for species observed in the samples.

* Epi-edaphic: surface-dwelling, hemi-edaphic: litter-dwelling, eu-edaphic: soil-dwelling. References: ^a^ (Berg et al. 1998); ^b^ (Ponge et al. 2006); ^c^ (Rusek & Josef 2007).

Reference

Berg MP, Kniese JP, Bedaux JJM, and Verhoef HA. 1998. Dynamics and stratification of functional groups of micro- and mesoarthropods in the organic layer of a Scots pine forest. *Biology & Fertility of Soils* 26:268-284.

Ponge J, Dubs F, Gillet S, Sousa J, and Lavelle P. 2006. Decreased biodiversity in soil springtail communities: the importance of dispersal and landuse history in heterogeneous landscapes. *Soil Biology and Biochemistry* 38:1158-1161. 10.1016/j.soilbio.2005.09.004

Rusek, and Josef. 2007. A new classification of Collembola and Protura life forms. Contributions to soil zoology in Central Europe II Proceedings of the 8th Central European Workshop on Soil Zoology. České Budějovice: ISB BC AS CR. p 109-115.
