# Supplementary material for: Marshland restoration benefits Collembola recruitment: a long-term chronosequence study in Sanjiang mire marshland, China
Source: PeerJ. 2019 Jun 27;7:e7198. doi: 10.7717/peerj.7198 (PMC6599674; doi:10.7717/peerj.7198)
Supplement: Supplemental Information 3 — 1. Body length: 1: very small (< 1 mm); 2: Small (from 1 to 2 mm); 3: Medium (from 2 to 3 mm); 4: Large (> 3 mm). 2. Reproduction type: 1: parthenogenetic; 2: sexual. 3. Dispersal: 1: slow; 2: fast. 4. Life form (Bokhorst et al., 2017): 1: eu-edaphic; 2: hemi-edaphic; 3: epi-edaphic. [file peerj-07-7198-s003.docx]

**Table S2.** Functional trait modalities of the collembolan species collected during the study.

| **Species name** | **Body length** | **Reproduction type** | | **Dispersal** | **Life form** |
| --- | --- | --- | --- | --- | --- |
| *Arrhopalites* sp.1 | 2 | *1* | *1* | | 1 |
| *Bourletiella* sp.1 | 2 | *2* | *2* | | 3 |
| *Bourletiella* sp.2 | 2 | *2* | *2* | | 3 |
| *Desoria* sp.1 | 2 | *2* | *2* | | 2 |
| *Desoria* sp.2 | 2 | *2* | *2* | | 2 |
| *Desoria* sp.3 | 3 | *2* | *2* | | 2 |
| *Desoria* sp.4 | 3 | *2* | *2* | | 2 |
| *Entomobrya* sp.1 | 2 | *2* | *2* | | 3 |
| *Entomobrya* sp.2 | 2 | *2* | *2* | | 3 |
| *Entomobrya* sp.3 | 2 | *2* | *2* | | 3 |
| *Entomobrya* sp.4 | 3 | *2* | *2* | | 3 |
| *Folsomia bidendata* | 2 | *2* | *1* | | 2 |
| *Folsomia sp.2* | 3 | *2* | *1* | | 2 |
| *Folsomia* sp.3 | 2 | *2* | *1* | | 2 |
| *Folsomides* sp.1 | 1 | *1* | *1* | | 3 |
| *Folsomides* sp.2 | 1 | *1* | *1* | | 3 |
| *Friesea* sp.1 | 2 | *2* | *1* | | 2 |
| *Hypogastrura* sp.1 | 2 | *2* | *1* | | 2 |
| *Hypogastrura* sp.2 | 2 | *2* | *1* | | 2 |
| *Hypogastrura* sp.3 | 2 | *2* | *1* | | 2 |
| *Isotomiella* sp.1 | 1 | *1* | *1* | | 1 |
| *Isotomodes* sp.1 | 1 | *1* | *1* | | 1 |
| *Lepidocyrtus felipei* | 4 | *2* | *2* | | 3 |
| *Allonychiurus songi* | 2 | *2* | *1* | | 1 |
| *Oligaphorura ursi* | 2 | *2* | *1* | | 1 |
| *Protaphorura armata* | 2 | *2* | *1* | | 1 |
| *Orchesellides sinensis* | 4 | *2* | *2* | | 3 |
| *Proisotoma* sp.1 | 2 | *1* | *1* | | 2 |
| *Ptenothrix.*sp.1 | 2 | *2* | *2* | | 3 |
| *Tomocerus nigrus* | 4 | *2* | *2* | | 3 |
| *Tullbergia* sp.1 | 2 | *1* | *1* | | 1 |

1. Body length: 1: very small (< 1 mm); 2: Small (from 1 to 2 mm); 3: Medium (from 2 to 3 mm); 4: Large (> 3 mm).
2. Reproduction type: 1: parthenogenetic; 2: sexual.
3. Dispersal: 1: slow; 2: fast.
4. Life form (Bokhorst et al. 2017)(Bokhorst, 2017 #506): 1: eu-edaphic; 2: hemi-edaphic; 3: epi-edaphic.

Reference

Bokhorst S, Berg MP, and Wardle DA. 2017. Micro-arthropod community responses to ecosystem retrogression in boreal forest. *Soil Biology & Biochemistry* 110:79-86.
