# Supplementary material for: Marshland restoration benefits Collembola recruitment: a long-term chronosequence study in Sanjiang mire marshland, China
Source: PeerJ. 2019 Jun 27;7:e7198. doi: 10.7717/peerj.7198 (PMC6599674; doi:10.7717/peerj.7198)
Supplement: Supplemental Information 4 — “+++” stands for dominant species, with individuals accounting for more than 10% of the total individuals; “++” stands for common species, with individuals accounting for 1% ∼ 10%; “+” stands for dominant species, with individuals accounting for less than 1%. [file peerj-07-7198-s004.docx]

| **Collembola** | | **Intact Marshland** | | **Cultivated Treatment** | | **Restored Treatments** | | | |
| --- | --- | --- | --- | --- | --- | --- | --- | --- | --- |
|  |  | **IM** | **Frequency (%)** | **CU15** | **Frequency (%)** | **RE06** | **Frequency (%)** | **RE12** | **Frequency (%)** |
| **Epi-edaphic** | *Bourletiella* sp. 1 | 65 | 0.80 (+) | 4 | 0.09 (+) | 34 | 0.66 (+) | 123 | 1.44 (++) |
|  | *Bourletiella* sp. 2 | 1 | 0.01 (+) | 0 | 0.00 (+) | 39 | 0.76 (+) | 26 | 0.30 (+) |
|  | *Entomobrya* sp. 1 | 0 | 0.00 (+) | 17 | 0.36 (+) | 37 | 0.72 (+) | 18 | 0.21 (+) |
|  | *Entomobrya* sp. 2 | 2 | 0.02 (+) | 0 | 0.00 (+) | 4 | 0.08 (+) | 1 | 0.01 (+) |
|  | *Entomobrya* sp. 3 | 4 | 0.05 (+) | 4 | 0.09 (+) | 0 | 0.00 (+) | 0 | 0.00 (+) |
|  | *Entomobrya* sp. 4 | 69 | 0.84 (+) | 18 | 0.39 (+) | 104 | 2.03 (++) | 135 | 1.58 (++) |
|  | *Folsomides* sp. 1 | 0 | 0.00 (+) | 10 | 0.21 (+) | 4 | 0.08 (+) | 86 | 1.01 (++) |
|  | *Folsomides* sp. 2 | 374 | 4.58 (++) | 420 | 9.00 (++) | 824 | 16.06 (+++) | 1558 | 18.25 (+++) |
|  | *Lepidocyrtus* *felipei* | 156 | 1.91 (++) | 1073 | 22.98 (+++) | 205 | 3.99 (++) | 231 | 2.71 (++) |
|  | *Orchesellides sinensis* | 98 | 1.20 (++) | 86 | 1.84 (++) | 14 | 0.27 (+) | 130 | 1.52 (++) |
|  | *Ptenothrix* sp. 1 | 17 | 0.21 (+) | 3 | 0.06 (+) | 18 | 0.35 (+) | 9 | 0.11 (+) |
|  | *Tomocerus* *nigrus* | 138 | 1.69 (++) | 0 | 0.00 (+) | 101 | 1.97 (++) | 59 | 0.69 (+) |
|  | **Total epi-edaphic** | 924 |  | 1635 |  | 1384 |  | 2376 |  |
| **Hemi-edaphic** | *Desoria* sp. 1 | 254 | 3.11 (++) | 17 | 0.36 (+) | 90 | 1.75 (++) | 243 | 2.85 (++) |
|  | *Desoria* sp. 2 | 142 | 1.74 (++) | 25 | 0.54 (+) | 139 | 2.71 (++) | 476 | 5.57 (++) |
|  | *Desoria* sp. 3 | 56 | 0.69 (+) | 0 | 0.00 (+) | 22 | 0.43 (+) | 112 | 1.31 (++) |
|  | *Desoria* sp. 4 | 305 | 3.73 (++) | 2 | 0.04 (+) | 196 | 3.82 (++) | 329 | 3.85 (++) |
|  | *Folsomia* *bidendata* | 587 | 7.18 (++) | 19 | 0.41 (+) | 2 | 0.04 (+) | 4 | 0.05 (+) |
|  | *Folsomia* sp. 2 | 52 | 0.64 (+) | 268 | 5.74 (++) | 383 | 7.46 (++) | 267 | 3.13 (++) |
|  | *Folsomia* sp. 3 | 1 | 0.01 (+) | 0 | 0.00 (+) | 25 | 0.49 (+) | 3 | 0.04 (+) |
|  | *Friesea* sp. 1 | 10 | 0.12 (+) | 7 | 0.15 (+) | 15 | 0.29 (+) | 6 | 0.07 (+) |
|  | *Hypogastrura* sp. 1 | 181 | 2.22 (++) | 33 | 0.71 (+) | 107 | 2.08 (++) | 183 | 2.14 (++) |
|  | *Hypogastrura* sp. 2 | 400 | 4.90 (++) | 24 | 0.51 (+) | 263 | 5.12 (++) | 419 | 4.91 (++) |
|  | *Hypogastrura* sp. 3 | 0 | 0.00 (+) | 0 | 0.00 (+) | 116 | 2.26 (++) | 11 | 0.13 (+) |
|  | *Proisotoma* sp. 1 | 28 | 0.34 (+) | 3 | 0.06 (+) | 48 | 0.94 (+) | 95 | 1.11 (++) |
|  | **Total hemi-edaphic** | 2016 |  | 398 |  | 1406 |  | 2148 |  |
| **Eu-edaphic** | *Arrhopalites* sp.1 | 322 | 3.94 (++) | 14 | 0.30 (+) | 50 | 0.97 (+) | 170 | 1.99 (++) |
|  | *Isotomiella* sp. 1 | 1061 | 12.98 (+++) | 112 | 2.40 (++) | 698 | 13.60 (+++) | 99 | 1.16 (++) |
|  | *Isotomodes* sp. 1 | 2 | 0.02 (+) | 1 | 0.02 (+) | 0 | 0.00 (+) | 0 | 0.00 (+) |
|  | *Allonychiurus songi* | 1721 | 21.06 (+++) | 1476 | 31.61 (+++) | 794 | 15.47 (+++) | 1372 | 16.07 (+++) |
|  | *Oligaphorura ursi* | 1019 | 12.47 (+++) | 624 | 13.36 (+++) | 442 | 8.61 (++) | 972 | 11.38 (+++) |
|  | *Protaphorura armata* | 1018 | 12.46 (+++) | 382 | 8.18 (++) | 358 | 6.98 (++) | 1402 | 16.42 (+++) |
|  | *Tullbergia* sp. 1 | 88 | 1.08 (++) | 27 | 0.58 (+) | 0 | 0.00 (+) | 0 | 0.00 (+) |
|  | **Total eu-edaphic** | 5231 |  | 2636 |  | 2342 |  | 4015 |  |

**Table S3.** Total number of Collembola of all sampling times across all sites. “+++” stands for dominant species, with individuals accounting for more than 10% of the total individuals; “++” stands for common species, with individuals accounting for 1% ~ 10%; “+” stands for dominant species, with individuals accounting for less than 1%.
