# Supplementary material for: Marshland restoration benefits Collembola recruitment: a long-term chronosequence study in Sanjiang mire marshland, China
Source: PeerJ. 2019 Jun 27;7:e7198. doi: 10.7717/peerj.7198 (PMC6599674; doi:10.7717/peerj.7198)
Supplement: Supplemental Information 5 — Capital letters indicate a significant effect among habitats and lowercase letters indicate a significant effect in the same habitat based on LSD test (repeated measurement ANOVA; P<0.05). Ind/m2: individuals/m2. [file peerj-07-7198-s005.docx]

|  |  | **IM** | **CU15** | **RE06** | **RE12** | ***F*-Value** | ***p*-Value** |
| --- | --- | --- | --- | --- | --- | --- | --- |
| Density (ind./m^2^) | Epi-edahpic | 1027±428^Cc^ | 1817±469^Bb^ | 1538±482^BCb^ | 2640±779^Ab^ | 7.24 | 0.001 |
|  | Hemi-edaphic | 2240±543^Ab^ | 442±172^Cc^ | 1562±508^Bb^ | 2387±536^Ab^ | 16.20 | <0.001 |
|  | Eu-edaphic | 5812±1250^Aa^ | 2929±957^Ba^ | 2602±709^Ba^ | 4461±893^Aa^ | 8.32 | <0.001 |
|  |  |  |  |  |  |  |  |

**Table S4.** Effects of marshland management on density (mean ± SE) of Collembola with three typical life-forms (Epi-, Hemi- and Eu-edaphic). Capital letters indicate a significant effect among habitats and lowercase letters indicate a significant effect in the same habitat based on LSD test (repeated measurement ANOVA; P<0.05). Ind/m^2^: individuals/m^2^.
